# Novel loci associated with resistance to downy and powdery mildew in grapevine

**DOI:** 10.3389/fpls.2024.1386225

**Published:** 2024-03-22

**Authors:** Valentina Ricciardi, Manna Crespan, Giuliana Maddalena, Daniele Migliaro, Lucio Brancadoro, David Maghradze, Osvaldo Failla, Silvia Laura Toffolatti, Gabriella De Lorenzis

**Affiliations:** ^1^ Dipartimento di Scienze Agrarie ed Ambientali, Università degli Studi di Milano, Milano, Italy; ^2^ Centro di Ricerca per la Viticoltura e l'Enologia, Consiglio per la ricerca in agricoltura e l'analisi dell'economia agraria (CREA), Conegliano, Italy; ^3^ Faculty of Viticulture-Winemaking, Caucasus International University, Tbilisi, Georgia; ^4^ Faculty of Agricultural Sciences and Biosystems Engineering, Georgian Technical University, Tbilisi, Georgia

**Keywords:** GWAS, SSR, SNP, phenotyping, *Rpv*, *Ren*, candidate genes

## Abstract

Among the main challenges in current viticulture, there is the increasing demand for sustainability in the protection from fungal diseases, such as downy mildew (DM) and powdery mildew (PM). Breeding disease-resistant grapevine varieties is a key strategy for better managing fungicide inputs. This study explores the diversity of grapevine germplasm (cultivated and wild) from Caucasus and neighboring areas to identify genotypes resistant to DM and PM, based on 13 Simple Sequence Repeat (SSR) loci and phenotypical (artificial pathogen inoculation) analysis, and to identify loci associated with DM and PM resistance, *via* Genome-Wide Association Analysis (GWAS) on Single Nucleotide Polymorphism (SNP) profiles. SSR analysis revealed resistant alleles for 16 out of 88 genotypes. Phenotypic data identified seven DM and 31 PM resistant genotypes. GWAS identified two new loci associated with DM resistance, located on chromosome 15 and 16 (designated as *Rpv36* and *Rpv37*), and two with PM resistance, located on chromosome 6 and 17 (designated as *Ren14* and *Ren15*). The four novel loci identified genomic regions rich in genes related to biotic stress response, such as genes involved in pathogen recognition, signal transduction and resistance response. This study highlights potential candidate genes associated with resistance to DM and PM, providing valuable insights for breeding programs for resistant varieties. To optimize their utilization, further functional characterization studies are recommended.

## 1 Introduction

*Vitis vinifera* L., the European grapevine, is a widely cultivated species native to the Eurasian region (Olmo et al., 1995). As of 2020, it was estimated that approximately 7.3 million hectares of arable land around the World were dedicated to grapevine cultivation, yielding a production of around 77.8 million tons of grapes. Only in Europe, the hectares are more than 3 million, with a production of around 27 million tons. Its production is mainly used for winemaking purposes (more than 50%), while less than 40% is reserved for consumption as table grapes, and less than 10% is used in producing raisins (https://www.oiv.int/public/medias/7909/oiv-state-of-the-world-vitivinicultural-sector-in-2020.pdf; https://www.fao.org/faostat/en/#data).

Grapevine is susceptible to several diseases, including downy mildew (DM) and powdery mildew (PM), which can cause significant economic losses in grapevine cultivation (Rienth et al., 2021). DM and PM are caused by the oomycete *Plasmopara viticola* (Berk. e Curt.) Berl. e De Toni, and the ascomycete *Erysiphe necator* Schw., respectively (Armijo et al., 2016).

Breeding of grapevine varieties with genetic resistance to diseases is a sustainable approach to reduce the use of plant protection chemical products. The identification of genetic loci associated with disease resistance has facilitated the development of new grapevine varieties with improved disease resistance, such as the PIWI varieties bred by some institutions in European Countries (Germany, France, Austria and Italy; https://www.internationalwinechallenge.com/Canopy-Articles/piwis-the-most-promising-varieties.html). The loci associated with DM resistance (*Rpv*) are 35 (Merdinoglu et al., 2003; Fischer et al., 2004; Wiedemann-Merdinoglu et al., 2006; Welter et al., 2007; Bellin et al., 2009; Marguerit et al., 2009; Blasi et al., 2011; Moreira et al., 2011; Schwander et al., 2012; Venuti et al., 2013; Ochssner et al., 2016; Divilov et al., 2018; Lin et al., 2019; Sapkota et al., 2019; Fu et al., 2020; Sargolzaei et al., 2020; Bhattarai et al., 2021; Zou et al., 2023). Most of them originate from North American and Asian wild *Vitis* species, and only three (*Rpv29-31*) originate from *vinifera* germplasm, coming from Caucasus (Sargolzaei et al., 2020). The loci associated with the PM resistance (*Ren* and *Run*) are 13 (Dalboí et al., 2001; Adam-Blondon et al., 2004; Barker et al., 2005; Welter et al., 2007; Hoffmann et al., 2008; Riaz et al., 2011; Blanc et al., 2012; Feechan et al., 2013; Pap et al., 2016; Zyprian et al., 2016; Teh et al., 2017; Zendler et al., 2017; Karn et al., 2021; Possamai et al., 2021), originate from North American and Asian wild *Vitis* species.

Genome-Wide Association Studies (GWAS) is a powerful method used to identify genetic loci (usually Single Nucleotide Polymorphism, SNP) that contribute to the control of complex traits in various organisms (Korte and Farlow, 2013). GWAS has been successfully used to identify genetic variants associated with many plant traits, such as yield, abiotic stress tolerance and disease resistance (Sakiroglu and Brummer, 2017; Kim and Reinke, 2019; Abdelraheem et al., 2021). In grapevine, it has been used to identify genetic markers associated with fruit quality (Laucou et al., 2018; Guo et al., 2019; Flutre et al., 2022), disease resistance (Zhang et al., 2017; Sargolzaei et al., 2020) and abiotic stress tolerance (Trenti et al., 2021).

Although European grapevine germplasm has been considered susceptible to DM and PM, some research has shown the opposite. For example, DM resistance genotypes have been identified in the germplasm of Caucasus, one of the two grapevine domestication centers (Dong et al., 2023), such as Mgaloblishvili and other genotypes (Bitsadze et al., 2015; Toffolatti et al., 2016, 2018). Likewise, Caucasian germplasm can boast genotypes resistant to PM, such as Kishmish vatkana, Shavtsitska and Tskhvedianis tetra (Hoffmann et al., 2008; Possamai et al., 2021). These discoveries make this germplasm a valuable genetic resource to breed disease-resistant grapevine varieties. This work aimed at exploring the potential of non-European *vinifera* germplasm for the breeding of *V. vinifera* varieties with improved resistance to *P. viticola* and *E. necator*, by identifying new loci, through a GWA approach.

## 2 Materials and methods

### 2.1 Plant material and DNA extraction

The grapevine accessions analyzed in this study accounted for 88 genotypes, wild (*V. vinifera* subsp. *sylvestris*) and cultivated (*V. vinifera* subsp. *sativa*), coming from Armenia, Azerbaijan, Georgia, Iran and Uzbekistan (**Supplementary Table 1**), grown in the germplasm collection of CREA - Research Center for Viticulture and Enology (located in Conegliano, Treviso, Italy, 45°51′07.6″N 12°15′28.6″E).

Young leaves were collected from each accession, sampled into 2 ml tubes and lyophilized. DNA was purified using DNeasy Plant Mini Kit (Qiagen, Hilden, Germany), following manufacturer’s instructions. DNA was quantified and quality checked with the NanoDrop™ One/One spectrofotomer (ThermoFisher Scientific, Waltham, Massachusetts, USA).

### 2.2 SSR genotyping

Genotyping was performed with 13 SSR markers, encompassing the nine internationally adopted (VVS2, VVMD5, VVMD7, VVMD25, VVMD27, VVMD28, VVDM32, VrZAG62, VrZAG79) (Maul et al., 2012), plus additional four (ISV2, ISV3, ISV4 and VMCNG4b9) (Crespan, 2003; Welter et al., 2007). Two multiplex PCRs combining 7 and 4 SSR loci (VVS2, VVMD7, VVMD28, VrZAG79, ISV2, ISV4 and VMC4B9; VVMD25, VVMD27, VVMD32 and VrZAG62) and one single PCR (for VVMD5 locus) were performed, in 10 µL final volume, as follow: 1x MyTaq muster mix (Bioline Reagents LTD, London, United Kingdom), primers (from 14 to 50 nM, depending by the primer), DNA (10 ng). Amplification conditions were: denaturation at 95°C for 3 min; followed by 35 cycles of 95°C for 30 sec, 57°C for 30 sec, 72°C for 30 sec; final elongation step 10°C ∞. 0.5 µL of PCR products were mixed with Genescan-500(LIZ) (Life Technologies, Foster City, CA, USA) as internal size standard and HI-DI formamide (Life Technologies), denatured and separated by capillary electrophoresis using an ABI 3130xl genetic analyzer (Life Technologies). Allele calling was performed with GeneMapper 4 (Life Technologies) software and a home-made bin-set built with reference varieties.

### 2.3 Genotyping for loci associated with resistance to DM and PM

All accessions were genotyped using SSR markers associated with known *Ren1*, *Rpv1*-*Run1* and *Ren3*-*Ren9* resistance loci to DM and PM (Possamai et al., 2021). Three multiplex PCR combining pairs of SSRs linked to resistance loci were performed. PCR mix, amplification conditions and capillary electrophoresis were as reported above, except for primers. Primer quantity varied from 7 to 14 nM (depending on the primer). Capillary electrophoresis has been performed as reported above. Allele calling was performed after building a bin-set with the reference varieties Artaban, Floreal, Vidoc, Voltis and Kishmish vatkana grown at Vivai Cooperativi Rauscendo repository (Rauscedo, Pordenone, Italy), and Regent from Consiglio per la Ricerca in Agricoltura e l’Analisi dell’Economia Agraria – Viticoltura ed Enologia collection in Susegana (Treviso, Italy).

### 2.4 SNP genotyping

The 88 samples were genotyped using the Vitis18kSNP genotyping array (Illumina Inc., San Diego, CA, United States), which includes 18,071 SNPs. Hybridization was carried out on 200 ng of genomic DNA at the laboratory of Fondazione Edmund Much (San Michele all’Adige, Trento, Italy). SNP data were filtered following these parameters, as reported in (De Lorenzis et al., 2015): i) samples showing a call quality value (p50GC) lower than 0.54 to be removed; ii) loci with a GenTrain (GT) score value lower than 0.6 to be removed; iii) samples with a marker missing rate > 20% to be removed; iv) loci with a minor allele frequency (MAF) < 5% to be removed.

### 2.5 Disease resistance evaluation

#### 2.5.1 Downy mildew assessment

Resistance to *P. viticola* was investigated following the protocol reported in (Sargolzaei et al., 2020). Briefly, naturally infected grapevine leaves were collected from vineyard plots not treated with fungicides acting against the downy mildew agent, located in Santa Maria della Versa (Pavia, Italy; North-Eastern Italian *P. viticola* population; 45°00’08.1”N 9°17’29.9”E) and Casarsa della Delizia (Pordenone, Italy; North-Western Italian *P. viticola* population; 45°56’45”N 12°47’18.8”E) (Maddalena et al., 2020). Sporangia were collected from the infected leaves and resuspended in distilled, sterile water to reach a 5x10^4^ sporangia mL^-1^ concentration. Three technical replicates constituted by leaf discs (1.5 cm in diameter), cut from three different leaves (biological replicates) collected from the 3^rd^–5^th^ leaf starting from the shoot apex of the plants, were sprayed with 1 mL of sporangia suspension by using an airbrush. The leaf discs, placed in Petri dishes (9 cm diameter) containing moistened filter paper and incubated at 22°C for 10 days, were then scored for the area covered by sporulation by using the OIV 452 resistance scoring method (OIV, n.d). Each leaf disc was assigned a class based on the extent of sporulation: i) Class 1 when the entire leaf disc surface was covered by sporulation; ii) Class 3 when most of the leaf disc surface was covered; iii) Class 5 when sporulation was limited to less than half of the leaf disc surface; iv) Class 7 when very few sporulating spots were visible; v) Class 9 when no sporulation was visible (Bove and Rossi, 2020). Downy mildew assessment was evaluated for three consecutive years (2013-2015). Phenotyping classification was carried out for three years on DM assessment data and only accessions with at least two years of data were considered for genotyping. The score “0” was attributed to susceptible accessions (OIV<7) and “1” to resistant accessions (OIV≥7).

#### 2.5.2 Powdery mildew assessment

Resistance to the powdery mildew agent, *E. necator*, was evaluated by artificially inoculating leaves coming from potted-grown cuttings in the greenhouse of the Department of Agricultural and Environmental Sciences of the University of Milan. Three leaves, starting from the third fully expanded leaf, from three replicate shoots per genotype were inoculated with *E. necator* conidia by gently touching them with an infected leaf (Fung et al., 2008). Infected leaves were collected from naturally infected plants grown in the same location, outside from the screenhouse. The inoculum used represented a mixed strain population of *E. necator* (Welter et al., 2017). The leaves were assessed for resistance using the OIV 455 scoring method (Bove and Rossi, 2020). Each leaf was assigned one of the following classes: i) Class 1, indicating leaves covered with unlimited patches of powdery mildew infection; ii) Class 3, denoting vast numbers of powdery mildew infection spots and abundant mycelium growth; iii) Class 5, representing patches of infection larger than 5 cm in diameter; iv) Class 7, limited patches of powdery mildew infection (< 2 cm); v) Class 9, indicating no disease symptoms or only a tiny visible spot (Riaz et al., 2011). An average OIV score was calculated for each accession. Powdery mildew assessment was evaluated for three consecutive years (2013-2015). Phenotyping classification was carried out for three years on PM assessment data and only accessions with at least two years of data were considered for genotyping. The score “0” was attributed to susceptible accessions (OIV<7) and “1” to resistant accessions (OIV≥7).

### 2.6 GWA analysis

The *poppr* package (Kamvar et al., 2014) for R software (R Core Team, 2021) was used to build a circular UPGMA (Unweighted Pair Group Method with Arithmetic Mean) phylogenetic tree, based on the Nei’s coefficient distance matrix. Principal Component Analysis (PCA) was performed in R using *adegenet* package (Jombart, 2008). The values of the first two components were plotted on a 2-D scatterplot. LEA package (Frichot and François, 2015) was used to perform structure analysis, varying the number of ancestral genetic groups (K) from 1 to 10. For each K value, ten repetition runs were performed. LEA cross-validation method was used to determine the most likely K value. The LD (linkage disequilibrium) estimation was evaluated by PLINK software (Purcell et al., 2007) using the following setting: –ld-window-r2 0, –ld-window 99999, –ld-window-kb 10000. The distances between loci were categorized into intervals of a fixed length (100 kb) and, for each interval, average r^2^ (Pearson’s squared correlation coefficient) was calculated. R was used to plot the average r^2^ values. LD decay was defined identifying the distance at which half of the maximum LD (LD_½,90_) has decayed (Vos et al., 2017).

GAPIT package (Wang and Zhang, 2021) for R was used to perform GWAS. The following algorithms were run: i) BLINK (Bayesian-information and Linkage-disequilibrium Iteratively Nested Keyway); ii) FarmCPU (Fixed and random model Circulating Probability Unification); iii) GLM (General Linear Model); iv) MLM (Mixed Linear Model). The first two PCs were estimated by GAPIT to assess the population structure and control the false marker-trait association. The *p*-value of each SNP was calculated, and only SNPs showing -log_10_(*p*) values higher than Bonferroni’s threshold were considered significantly associated with the resistance. The GWA algorithm performances were evaluated through quantile-quantile (QQ) plots.

Significant SNP loci were identified on both grapevine PN40024 v4 reference genome (Velt et al., 2023) and Mgaloblishvili genome (Ricciardi et al., 2023) to identify putative genes related to DM and PM resistance traits. SNP probes identifying the GWA signals were mapped against both genomes using BLASTn-short (Altschul et al., 1990). Based on LD_½,90_ (r^2 ^= 0.05) values, the annotated genes in the regions 0.5 Mb up-stream and down-stream of SNP probe location were identified as the putative candidate genes likely involved in disease resistance. To avoid possible annotation mistakes, the genes of each locus in the reference genome were mapped against Mgaloblishvili genome haplotypes and *viceversa* using GMAP (Wu and Watanabe, 2005). In PN40024 v4, the probes were found to have shifted in their positions, with instances (such as the be_C_T_chr17_16451257 SNP on chromosome 17) exhibiting shifts of up to 4 Mb. This insight proved instrumental in accurately extracting information regarding the gene content within regions linked to GWAS signals. To ensure comparability with previously reported loci, information from PN40024 12X v2 was also incorporated. This approach facilitated the maintenance of data comparability when assessing the positioning of GWAS signals in relation to previously documented loci.

## 3 Results

### 3.1 Varietal identification and screening for resistance loci

DNA analysis with the set of 13 SSR markers revealed 88 non redundant (**Supplementary Table 1**). After comparing these profiles with the ones reported in the *Vitis* International Variety Catalogue molecular database, 54 profiles were identified as new. Absheron Gelinbarmaghy B. (G4) showed to be triallelic at VVMD5 locus (**Supplementary Table 1**).

Regarding the presence of already known sources of resistance to DM and PM, no positive results were obtained for *Rpv1*-*Run1* and *Ren3*-*Ren9* loci. For *Ren1* locus, the allelic combination associated with the resistance identified in Kishmish vatkana was found in two genotypes, Tchumuta and Nakhiduri 04, while the one identified in Shavtsitska and in Tskhvedianis tetra was detected in 14 genotypes (Aladasturi, Alexandrouli, Atcharuli tetri, Barisakho turning 01, Chachkhriala – 01, Chkhaveri, Dzvelshavi, Kakhet, Khupishij, Kvelouri, Samebis seri 02, Seura, Skra 01, Tushis tbebi 02, six of them are wild genotypes). Interestingly, in the Georgian *sylvestris* Barisakho turning 01, the resistance appears to be in a homozygous state, unless for the presence of null allele profile (**Supplementary Table 1**).

### 3.2 SNP genetic diversity

**Figure 1:**
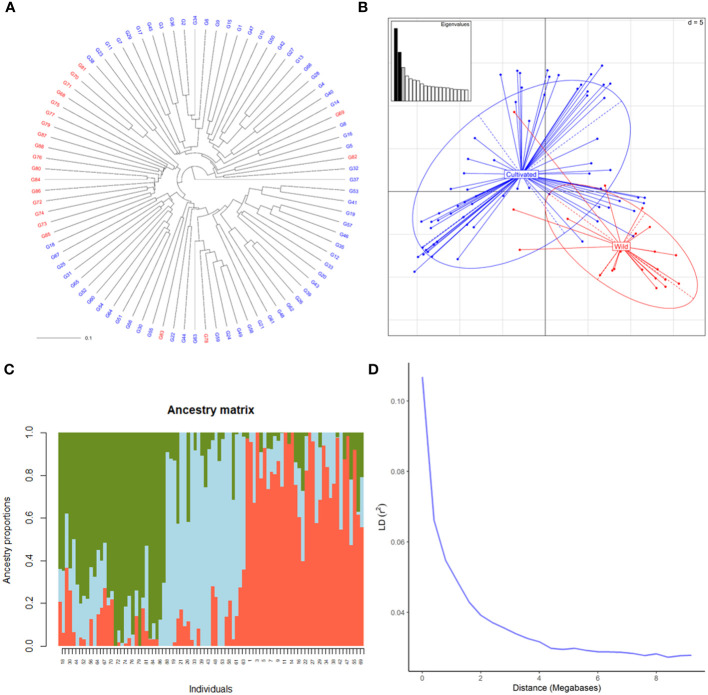
Genetic diversity of 88 cultivated and wild grapevine accessions, coming from Caucasus, Iran and Uzbekistan, genotyped using the Vitis18kSNP array. **(A)** UPGMA dendrogram showing relationships among cultivated (blue) and wild (red) individuals. **(B)** Scatterplot relationships among cultivated (blue) and wild (red) individuals, as represented by the first two principal components (PC1 along the horizontal axis, PC2 along the vertical axis) of PCA. **(C)** Admixture proportions as estimated by LEA package at K = 3, displayed in a barplot. Each sample is represented as a vertical bar, reflecting assignment probabilities to each of the three groups. **(D)** Decay of average linkage disequilibrium (LD r2) over distance (Mb).

SNP profiles obtained by the hybridization of DNA with the Vitis18kSNP genotyping array after filtering accounted for 13,213 loci (**Supplementary Table 2**). The filtered profiles were used to assess the genetic diversity of plant material, performing clustering analysis, PCA and structure analysis. Cluster analysis clearly discriminated between wild and cultivated samples (**Figure 1A**). Only four genotypes labelled as wild samples were clustered together with cultivated ones. Similarly, PCA discriminated between wild and cultivated samples, with some wild genotypes grouped together with cultivated ones (**Figure 1B**). The first two principal components (PC) accounted for 8 and 6% of genetic variability, respectively for first and second PC. The two compartments were mainly discriminated along PC1. According to the cross-validation plot, structure analysis identified K = 3, as the most likely number of ancestral populations, two for cultivated individuals (groups 1 and 2) and one for wild ones (**Figure 1C**). The percentage of admixed genotypes (with a membership probability < 80%) was 50% (**Supplementary Table 3**). Approximately half of both the cultivated and wild accessions were admixed (51 and 53%, respectively). As expected, LD decreased with the increase in physical distance between marker loci (**Figure 1D**). LD_½,90_ (r^2^ = 0.05) value was observed at around 0.9 Mb.

### 3.3 Disease resistance

Phenotypic data allowed us to identify DM and PM resistant varieties among the analyzed population. In the end, seven DM (OIV score ≥ 7) and 25 PM resistant varieties (OIV score ≥ 7) were identified (**Supplementary Table 4**). Most of the resistant accessions (30) were identified within *V. vinifera* subsp. *sativa*. However, three DM resistant accessions (Tushis tbebi 02, Chachkhriala - 01, Barisakho turning 01) and four PM resistant accessions (Ninotsminda - 13, Tedotsminda 25, Wild grape (the male flower) N2, and Skra 01) belong to *V. vinifera* subsp. *sylvestris*. All DM-resistant accessions originated from Georgia. The PM resistant accessions mainly belonged to Georgia (20 accessions) and Azerbaijan (8 accessions). Two PM resistant accessions were also found in the Armenian germplasm, and one in the material from Uzbekistan. Notably, an accession from Georgia, Tsirkvalis tetri (*V. vinifera* subsp. *sativa*), consistently exhibited high levels of resistance to PM (OIV score=9) and a good level of resistance to DM (OIV score=7).

### 3.4 Loci associated with DM and PM resistance

Four different statistical models (BLINK, FarmCPU, MLM and GLM) were tested to detect the loci associated with DM e PM resistance. Because PCA was able to capture the differences among the genotypes better than structure analysis, the values of the first two PCs were used as covariate in the GWA analysis.

**Figure 2:**
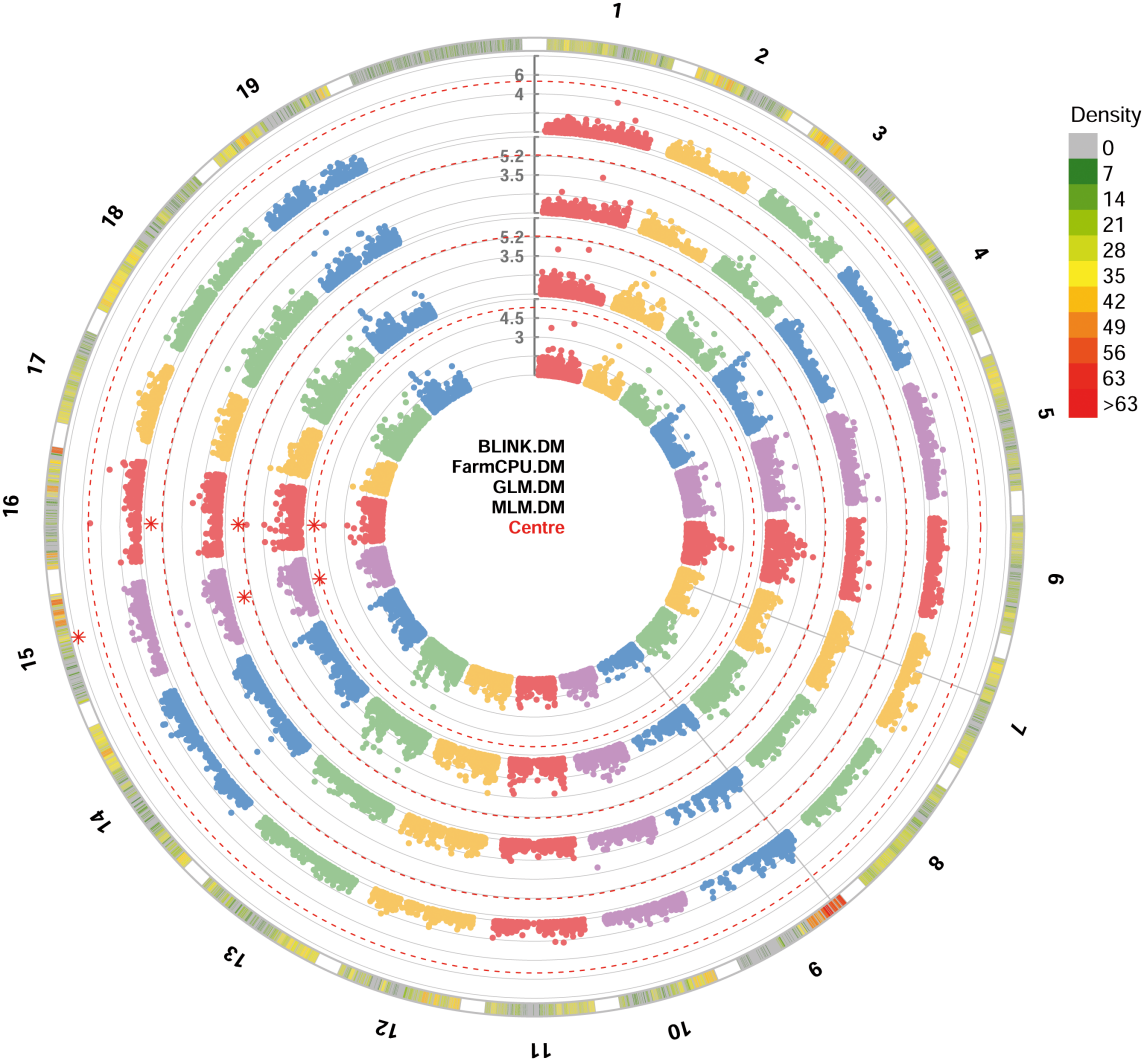
Circular Manhattan plot of -log10 p-values estimated for binary (resistant *vs*. susceptible) coded phenotypic response to downy mildew (DM) in a panel of 88 cultivated and wild grapevine accessions genotyped by 18 k SNPs and subjected to GWAS. Significant SNPs are highlighted with a star above the Bonferroni-adjusted threshold (red dotted line). Association analysis results of BLINK, FarmCPU, MLM and GLM (from the outer to the inner circle) models from GAPIT.

All the four GWAS models allowed to identify at least one significant SNP locus associated with DM resistance, showing -log_10_(*p*) values higher than Bonferroni’s threshold. All the significant SNP loci were at least detected by two models. BLINK and FarmCPU identified one locus each, while GLM and MLM models identified two loci each. These loci are, according to the information provided by Illumina: i) mu_s_G_A_chr15_11771804 SNP located in the chromosome 15 at position 11,771,804, identified by BLINK, GLM and MLM models; ii) chr16_8343864_A_G SNP located in the chromosome 16 at position 8,343,864, identified by FarmCPU, GLM and MLM models (**Figure 2**). *p*-values ranged from 1.84^e-8^, for mu_s_G_A_chr15_11771804 locus identified under the BLINK model to, 4.19^e-6^, for chr16_8343864_A_G locus identified under the MLM model (**Table 1**). QQ-plot showed that all the four models used in GWAS were able to account for population structure (**Supplementary Figure 1**). The phenotypic variance explained by the two detected SNP loci ranged from 18%, for chr16_8343864_A_G locus identified under the GLM and MLM models, to 81%, for mu_s_G_A_chr15_11771804 locus identified under the BLINK model (**Table 1**).

**Table 1:** List of significant SNP loci associated with downy and powdery mildew resistance.

SNP ID	Chromosome	Position (bp)	*p*-value	Phenotype variance explained (%)	GWAS model
Downy mildew					
mu_s_G_A_chr15_11771804	15	11,771,804	1.84e-08	80.6	BLINK
			9.92e-07	43.5	GLM
			3.78e-06	43.5	MLM
chr16_8343864_A_G	16	8,343,864	4.56e-07	65.6	FarmCPU
			1.59e-06	18.5	GLM
			4.19e-06	18.5	MLM
Powdery mildew					
chr6_18189932_G_T	6	18,189,932	6.73e-08	11.4	BLINK
			3.94e-07	7.75	GLM
			2.00e-06	11.4	MLM
be_C_T_chr17_16451257	17	16,451,257	1.63e-07	14.0	BLINK
			2.02e-07	7.72	GLM
			4.88e-07	14.1	MLM

Per each SNP, chromosome, position (PN40024 v4 genome), p-value, phenotype variance explained and GWAS models are reported.

**Figure 3:**
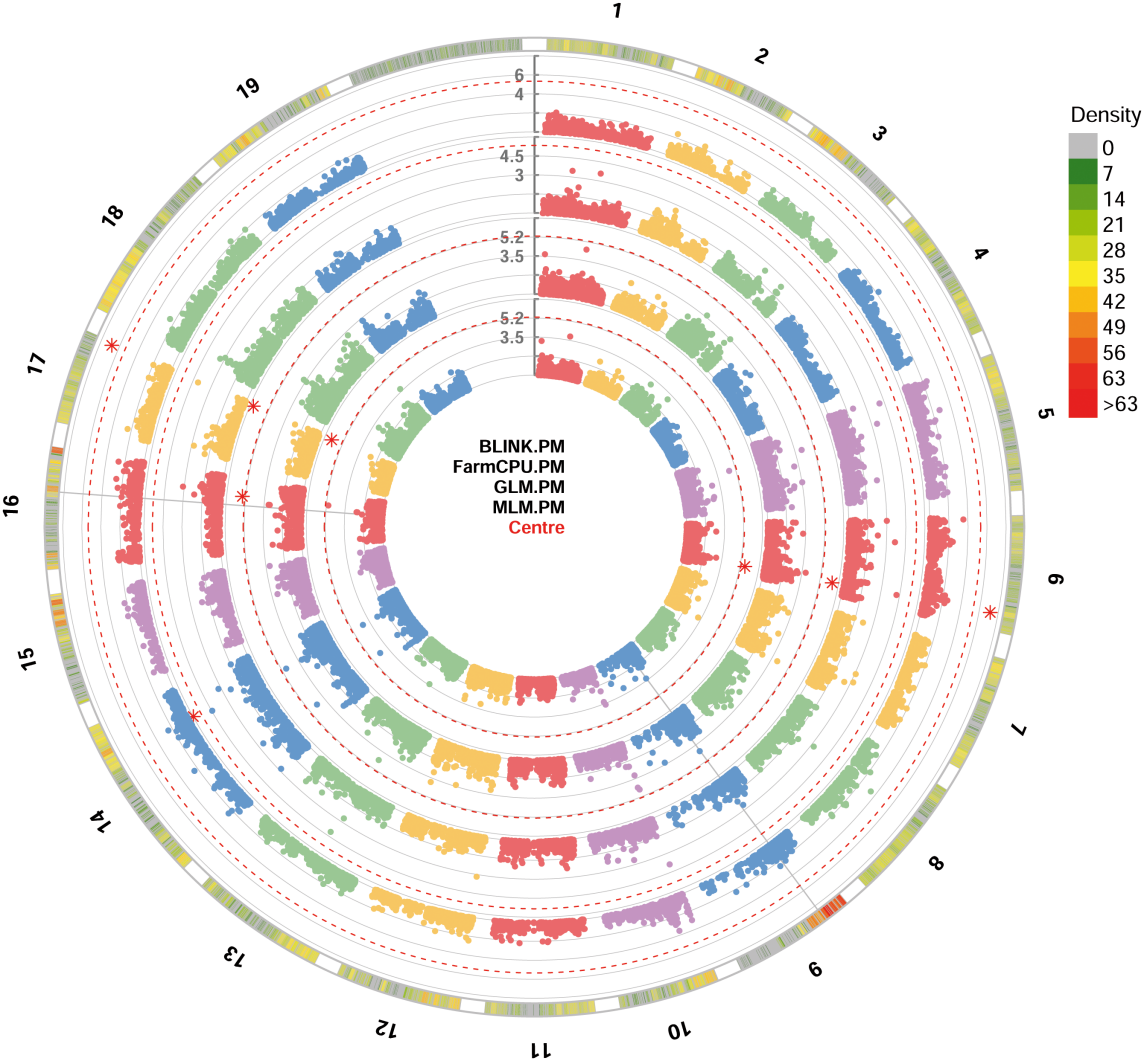
Circular Manhattan plot of -log10 p-values estimated for binary (resistant *vs*. susceptible) coded phenotypic response to powdery mildew (PM) in a panel of 88 cultivated and wild grapevine accessions genotyped by 18 k SNPs and subjected to GWAS. Significant SNPs are highlighted with a star above the Bonferroni-adjusted threshold (red dotted line). Association analysis results of BLINK, FarmCPU, MLM and GLM (from the outer to the inner circle) models from GAPIT.

For PM resistance, all the four GWAS models were able to identify significant SNP loci associated with the resistance, showing -log_10_(*p*) values higher than Bonferroni’s threshold. Since some of these loci were identified by only a model, only loci identified by at least two models were considered. These loci identified with BLINK, GLM and MLM models are: i) chr6_18189932_G_T SNP located in the chromosome 6 at position 18,189,932; ii) be_C_T_chr17_16451257 SNP located in the chromosome 17 at position 16,451,257 (**Figure 3**). *p*-values ranged from 6.73^e-8^, for chr6_18189932_G_T SNP locus identified under the BLINK model, to 2.00^e-6^, for chr6_18189932_G_T SNP locus identified under the MLM model (**Table 1**). QQ-plot showed that all the models used in GWAS were able to account for population structure, except for the GLM model (**Supplementary Figure 2**). The phenotypic variance explained by the two detected SNP loci ranged from 7.7%, for be_C_T_chr17_16451257 locus identified under the GLM model, to 14%, for be_C_T_chr17_16451257 locus identified under the BLINK model (**Table 1**).

### 3.5 Candidate gene prediction

In PN40024, 43 genes were found in the surrounding regions of the mu_s_G_A_chr15_11771804 SNP (chromosome 15), while in Mgaloblishvili genome, 41 genes were identified in haplotype 1 and 44 in haplotype 2 (**Supplementary Table 5**). The region seemed to be relatively conserved between PN40024 and Mgaloblishvili. Among the conserved genes between the two genomes, there were: ankyrin repeat family proteins (one gene in PN40024, three genes in both Mgaloblishvili haplotypes), a WRKY transcriptional factor 12, some phospholipases A DAD1 (defective in anther dehiscence 1) genes (three genes in PN40024 and Mgaloblishvili haplotype 2, and two genes in Mgaloblishvili haplotype 1), a serine/threonine-protein kinase D6PK-like and different ethylene-responsive transcription factors 1A – like (four genes in PN40024 and Mgaloblishvili haplotypes) (**Supplementary Table 5**). A cytochrome P450 78A5 – like gene and a VASCULAR ASSOCIATED DEATH 1 protein were present only in the Mgaloblishvili haplotypes (**Supplementary Table 5**).

The surrounding regions of the chr16_8343864_A_G SNP (chromosome 16) were more diverse in the two genomes. The gene content exhibited substantial variability even between the Mgaloblishvili haplotypes (**Supplementary Table 5**). In PN40024, 44 genes were identified, while, 32 and 45 genes were found in Mgaloblishvili haplotype 1 and 2, respectively. Some conserved genes were: receptors-like proteins, a rust resistance kinase Lr10-like, a ceramide kinase and a derlin-2 protein gene (**Supplementary Table 5**). Nevertheless, the gene content between the two genomes was very dissimilar, with Mgaloblishvili possessing more genes related to biotic stress response compared to PN40024. Examples of these genes are an F-box/kelch-repeat plant protein, two WD-repeat-containing proteins, an armadillo/beta-catenin repeat family protein gene, a subtilase-like protease gene, and a ubiquinol-cytochrome-c reductase complex assembly factor 1. Interestingly, Mgaloblishvili haplotype 2 carried also a MLO family protein gene and a Cf-4/9 disease resistance-like family protein gene, together with a high number of MYB transcription factors (**Supplementary Table 5**).

The surrounding regions of the chr6_18189932_G_T SNP (chromosome 6) were denser in gene content compared to the other loci. PN40024 holds 105 genes and Mgaloblishvili 85 and 91 genes, respectively for haplotype 1 and 2 (**Supplementary Table 5**). The gene content varied greatly in terms of performed function, nevertheless most genes were found to be directly or indirectly involved in biotic stress responses, such as some serine kinases, genes related to the production of secondary metabolites (i.e. (+)-neomenthol dehydrogenase-like genes and 4-coumarate–CoA ligase-like 7 genes), some proteases genes, genes involved in auxins signaling pathway and some transporters (**Supplementary Table 5**).

Fifty-one genes were found in the surrounding regions of the be_C_T_chr17_16451257 SNP (chromosome 17) for PN40024, and 42 and 41 for Mgaloblishvili haplotype 1 and 2, respectively (**Supplementary Table 5**). In this case, the genes and their functions were more conserved between the two genomes. For the majority of genes, functions appeared to be related to receptors and signaling pathways, plant core metabolism and, interestingly, to abiotic stress responses (**Supplementary Table 5).** Some of those genes, detected in both PN40024 and Mgaloblishvili were: a proteasome subunit *alpha* type-1-A-like, a putative LRR receptor-like serine/threonine-protein kinase gene, a COP9 signalosome complex subunit 2 gene and ubiquitin-like-specific protease ESD4 gene.

## 4 Discussion

### 4.1 *V. vinifera* germplasm is a source of resistance to DM and PM

The identification of resistant varieties with a *vinifera* background could be a promising solution to simplify breeding programs of resistant varieties and achieve a sustainable management of viticulture. By pinpointing the specific genetic regions in grapevines associated with resistance to these pathogens, researchers and viticulturists can develop targeted breeding programs to cultivate grape varieties with built-in resistance. Traditionally, *V. vinifera* is considered susceptible to DM and PM. Recently, several resistant genotypes have been identified in *vinifera* germplasm, such as Mgaloblishvili and other Caucasian accessions (Bitsadze et al., 2015; Toffolatti et al., 2016; Margaryan et al., 2023), Kishmish Vatkana from Central Asia (Hoffmann et al., 2008) and some European wild genotypes (Lukšić et al., 2022). In this work, 88 genotypes from wild and cultivated grapevine compartments were investigated to identify individuals resistant to DM and PM. In accordance with the literature, the phenotypic characterization revealed that the majority (92% and 63%) of the tested *V. vinifera* accessions had high levels of susceptibility to the DM and PM agents. This aligns with the typical description for the species, which did not co-evolve with the pathogens (Fontaine et al., 2021). Seven of the 88 accessions screened here were found out to be resistant to DM (four *sativa* and three *sylvestris*), including the previously described Mgaloblishvili accession (Toffolatti et al., 2016, 2018, 2020), and 31 were resistant to PM (four *sylvestris*), including the well-known Kishmish Vatkana and a high number of Georgian individuals. This discovery opens new perspectives on genetic improvement of the *V. vinifera* species for disease resistance. Traditionally, resistance traits are introgressed in *V. vinifera* following crossings with American and Asian species. However, analyzing the list of resistant varieties published on the Vitis International Variety Catalogue - VIVC website (www.vivc.de, “Genetic resources monitoring” and “Resistance loci/varieties” lists of database search. Access date: 7/11/2023), it is observed that the range of QTL employed in genetic improvement is limited to *Rpv3*, *Rpv10*, and *Rpv12* for DM, and *Run3* and *Run9* for PM. The disease protection conferred by resistance genes can be easily overcome by pathogens. For example, *P. viticola* strains have shown the ability to overcome resistance traits in varieties like Bianca (carrying *Rpv3* locus) and Regent (possessing the *Rpv3-1* QTL) (Toffolatti et al., 2012; Delmotte et al., 2014; Eisenmann et al., 2019). The *E. necator* Musc4 isolate collected from *V. rotundifolia* also exhibited virulence on *Run1* accessions. Pyramiding dominant and recessive genes in grapevine is a strategy to increase the durability potential of resistant varieties (Merdinoglu et al., 2018). In this context, the discovery and exploitation of *V. vinifera* resistance genes could contribute to increase the resilience of resistant varieties.

Based on the SSR data predictions associated with *Ren1* locus, it was anticipated that 17 varieties would exhibit resistance to PM, comprising 11 *sativa* and six *sylvestris* individuals. However, phenotypic data confirmed PM resistance in only 10 genotypes (58.8%), with nine *sativa* and one *sylvestris*. The current data suggest that the *Ren1* analysis system is effective for *sativa* samples, albeit not entirely reliable, while proving unreliable for *sylvestris* ones. SC8–0071-014 and SC47_20, the SSR markers utilized for *Ren1* locus tracing, are situated on PN40024 chromosome 13 at 16.87 and 18.24 Mb, respectively (Possamai et al., 2021). Consequently, their reciprocal distance is approximately 1.37 Mb, a value exceeding the LD calculated for grapevine (0.9 Mb in this study). It can be hypothesized that if *Ren1* was inherited from *sylvestris*, the allelic pairs crucial for *Ren1* selection might have been established subsequently through recombination, coupling the beneficial alleles with this resistance locus.

### 4.2 New SNP loci associated with DM and PM resistance

To date, 35 QTLs conferring resistance to DM and 13 QTLs conferring resistance to PM have been identified within *Vitis* spp. and *V. vinifera.* Despite the high number of loci, very few of them are characterized to gene level: *Rpv1* (Feechan et al., 2013), *Rpv3* (Foria et al., 2018), *Rpv33* (Zou et al., 2023), *Ren2*, *Ren3*, *Ren4*, *Ren6*, *Ren11*, *Ren12* and *Run1* (Massonnet et al., 2022; Sapkota et al., 2023). The limited accessibility of complete grapevine genomes in previous years, coupled with the challenges associated with tracking population evolution in grapevine because of its high heterozygosity and perennial life cycle, likely account for this deficiency (Shi et al., 2023). To mitigate the occurrence of some of these challenges, in this work the SNP probe location data (based on PN40024 12X v2) were used to obtain information about their location in the newest version of PN40024 genome (v4).

All the four significant SNPs identified in this work did not co-localize with previously known loci. Nonetheless, it was interesting to notice the proximity of mu_s_G_A_chr15_11771804 locus (chromosome 15, 11.7 Mb in PN40024 12x v2, 13.1 Mb in PN40024 v4), associated with *P. viticola* resistance, with *Ren3* (associated to *E. necator* resistance) location in the old version of PN40024 genome (12x v2) (Zendler et al., 2017). This observation may imply an evolutionary adaptation specific to that chromosomal region. The chr16_8343864_A_G locus is located on chromosome 16 (8.3 Mb in PN40024 12x v2 and 7.2 Mb in PN40024 v4). According to literature, *Rpv31* (Sargolzaei et al., 2020) is also located on chromosome 16, but its position was identified to encompass the region between 22.09 and 22.28 Mb (Ricciardi et al., 2023), not in linkage with chr16_8343864_A_G locus. In summary, the absence of physical co-location between the SNPs identified in this study and the previously identified QTLs leads to the conclusion that the four loci represent novel associations. Following the pre-existing numbering scheme, the locus mu_s_G_A_chr15_11771804 (chromosome 15) is designated as *Rpv36*, the locus chr16_8343864_A_G (chromosome 16) is designated as *Rpv37*, the locus chr6_18189932_G_T (chromosome 6) is designated as *Ren14* and the locus be_C_T_chr17_16451257 (chromosome 17) is designated as *Ren15*.

### 4.3 PN40024 and Mgaloblishvili genomes differ in new DM and PM resistance loci

To assure the most accurate content prediction for the genomic regions identified in this work, the SNP probes on both PN40024 versions (v2 and v4) and Mgaloblishvili genome (Ricciardi et al., 2023) were mapped. In the two genomes, the gene content was conserved with some differences. The most conserved locus was *Rpv36* locus for the resistance to DM, while the most different was *Ren14* locus for the resistance to PM. Frequently, one of the two haplotypes of Mgaloblishvili showed a more similar content to PN40024 genome, suggesting a greater genetic proximity to the reference, in contrast to the other haplotype, which exhibited significant variability (Magris et al., 2021). This difference may be attributed to structural variation in the locus between the compared genomes. If this proves to be accurate, the difference could be interpreted as a more substantial genetic distance between PN40024 and the highly different Mgaloblishvili haplotypes. Moreover, it might indicate a significant level of differentiation in these regions among *V. vinifera* varieties. This differentiation could potentially contribute to elucidating the existing variability in the distribution of PM resistance phenotypes within *V. vinifera* populations.

### 4.4 Regions associated with DM and PM resistance are related to both biotic and abiotic stress response

Plants have evolved various mechanisms to defend themselves against biotic stress, which includes threats from pathogens and herbivores. Some of the key defense mechanisms include: hormonal signaling, physical barriers, chemical defense. These mechanisms are part of a complex and dynamic interplay between plants and their biotic environment, allowing them to adapt and defend themselves against a wide range of stressors (Iqbal et al., 2021). In this work, both genomes revealed that the regions associated with DM and PM resistance contained at least a core of genes related to biotic stress responses. A greater abundance of genes specifically associated with plant defense against biotic stress was identified in Mgaloblishvili in comparison to PN40024. Nevertheless, also PN40024 showed at least a core of genes involved in the same functions, such as genes related to perceive pathogen signal [receptor-like proteins; (Yang et al., 2012)], signal transduction[ethylene-responsive transcription factors 2; (Licausi et al., 2013)] and microbial compounds [(+)-neomenthol dehydrogenase-like; (Munhoz et al., 2015)].

It is noteworthy that certain genes identified in this study are associated with both biotic and abiotic stress responses. For instance, transporter genes and COP9 (constitutive photomorphogenesis 9) signalosome complex subunit-like protein genes were found in both the genomic regions associated with resistance to DM, on chromosome 15, and to PM, on chromosome 17. Importantly, these genes exhibit conservation across both the PN40024 and Mgaloblishvili genomes. COP9 signalosome complex subunit-like protein genes are involved in the regulation of development and hormones, and in response against pathogens, like Tobacco mosaic virus and *Botrytis cinerea*, and abiotic stresses, such as oxidative stress and salt stress (Hind et al., 2011; Stratmann and Gusmaroli, 2012; Tan et al., 2016; Wei et al., 2018). On the chromosome 17 region, NAC domain-containing protein 86-like, in both genomes, and scarecrow-like protein 23, in Mgaloblishvili haplotype 1, are genes associated with biotic and abiotic response as well. NAC domain-containing protein genes are recognized for their involvement in responses to various pathogens, including *Pseudomonas syringae*, *B*. *cinerea*, *Alternaria brassicola* and *Puccinia striiformis*, as well as diverse abiotic stresses like drought and salt stress (Xia et al., 2010; Tariq et al., 2022). Scarecrow-like proteins, associated with abiotic stress responses, operate through auxin and gibberellin signaling pathways (Wang et al., 2020).

### 4.5 Regions associated with DM and PM resistance are rich in genes related to resistance mechanism

Regarding regions associated with DM, the *Rpv36* locus revealed a highly conserved region between the genomes of PN40024 and Mgaloblishvili. Among the conserved genes shared by the two genomes, the majority appeared to be implicated in pathogen response, with some forming clusters. Particularly notable were the ankyrin repeat family protein genes, which were in Mgaloblishvili in three copies, contrasting with PN40024 single copy. As per the literature, these genes function as receptor-like proteins and play a regulatory role in plant immunity (Yang et al., 2012). Other receptors included the serine/threonine-protein kinase genes, which are currently recognized as well-established contributors to the defense response against various pathogens, ranging from fungi (thus causing PM) to viruses (Cao et al., 2011). These genes were found in equivalent copy numbers (two) in the genomes of both PN40024 and Mgaloblishvili haplotypes. Closely associated with these genes is another cluster identified in this region, conserved between the two genomes, the phospholipase A DAD1 genes. Involved in lipid metabolism and activated in response to pathogen infections, these genes are crucial components in the mechanisms associated with plant immunity (Lee and Park, 2019). In grapevine, there is evidence that grapevine resistance to DM could be mediated by lipid associated signaling (Laureano et al., 2018). These genes were found in equivalent copy numbers in the genomes of both PN40024 and Mgaloblishvili haplotype 2 (three copies each), whereas there is a discrepancy in Mgaloblishvili haplotype 1 genome, where two copies were found. Interestingly, this region holds numerous transcription factors, such as WRKY transcription factor 12 gene, whose overexpression results in the reduction of soft rot symptoms in *Arabidopsis thaliana* and Chinese cabbage (Laureano et al., 2018), and a cluster of ethylene-responsive transcription factors 2 gene, notoriously involved in the regulation of defense responses in many plant species (Preí et al., 2008; Licausi et al., 2013). These particular genes were found in an equivalent number of copies in both PN40024 and Mgaloblishvili genomes. In contrast to what was observed for the PN40024 genome, the gene content in Mgaloblishvili appeared to be even more specialized towards the defense response against pathogens. Notably, there were genes such as a cytochrome P450 78A5-like gene, implicated in the production of secondary metabolites in response to stresses (Chakraborty et al., 2023), and the VASCULAR ASSOCIATED DEATH 1 gene. The latter functions as a regulator of cell death and it is involved in defense responses in vascular tissues against pathogens such as *P. syringae* (Lorrain et al., 2004).

The region identified by *Rpv37* locus exhibited significant variations among the two genomes. Many genes were receptors-like proteins, such as a kinase Lr10-like genes, involved in resistance to rust disease in wheat (Feuillet et al., 2003). In a prior study investigating the transcriptome of Mgaloblishvili during *P. viticola* infection, the variety exhibited upregulation of several Lr10-like kinase genes following inoculation with the pathogen (Toffolatti et al., 2018). In general, most genes related to pathogens defense was found in the Mgaloblishvili genome. Among the most interesting genes, it has been found a F-box/kelch-repeat plant gene and subtilase-like protease gene. Surprisingly, the first gene is involved in the tolerance against PM in grapevine. It is a component of the E3 ubiquitin ligase complex (whose components were found in the regions identified in this work), and its overexpression leads to enhanced tolerance against the pathogen through the proteasome system pathway (Wang et al., 2017). The second gene, subtilase-like protease, is known to be involved in the response against both *P. viticola* and *E. necator*, by contributing to the successful establishment of resistance response (Figueiredo et al., 2018). The genes were detected in *Rpv31* locus of the Mgaloblishvili genome as well (Ricciardi et al., 2023), confirming their important role in the defense against these pathogens. Within this region, the two Mgaloblishvili haplotype genome exhibited substantial dissimilarity, with notable significance in haplotype 2 due to the presence of an MLO family protein gene and a Cf-4/9 disease resistance-like family protein gene. MLO genes are a well-known susceptibility gene to PM (Pessina et al., 2016), while Cf-4/9 disease resistance-like family proteins are known to confer resistance to *Cladosporium fulvum* in tomato (Kruijt et al., 2004).

The regions associated with PM resistance showed lower conservation between PN40024 and Mgaloblishvili genome. The first locus, *Ren14*, pinpointed a region characterized by significantly higher gene density compared to the others (105 genes in PN40024 genome, 85 and 91 genes in Mgaloblishvili haplotype 1 and haplotype 2, respectively). Despite the great variability in the performed functions, most genes were found to be involved in biotic stress responses. Among those, some serine kinases and some genes involved in the production of secondary metabolites for defense purposes, such as (+)-neomenthol dehydrogenase-like and 4-coumarate–CoA ligase genes, were discovered. The (+)-neomenthol dehydrogenase is a defense antimicrobial protein that is involved in the neomenthol biosynthesis induced in response to pathogen attack (Munhoz et al., 2015). These findings corroborate previous studies on the defense mechanisms of Mgaloblishvili. It has been demonstrated that this variety produces secondary metabolites, including volatile organic compounds (VOCs), in response to *P. viticola* infection. This highlights the pivotal role of this mechanism in the variety defense strategy (Toffolatti et al., 2018; Ricciardi et al., 2021). The second gene synthesizes 4-coumarate–CoA ligase. This enzyme is known to be involved in the cotton plant resistance to *Verticillium dahliae*, by promoting vascular lignification and metabolic flux through jasmonic acid signaling pathway (Alariqi et al., 2023). Other R-genes within this region were found to participate in the auxin signaling pathway, well-documented for its involvement in biotic stress responses (Kazan and Manners, 2009; Kunkel and Harper, 2018), as well as in the transport of nutrients and compounds.

Similar to other analyzed regions, the *Ren15* locus identified a region rich in genes associated with pathogen responses. This includes various receptors, such as LRR receptor-like serine/threonine-protein kinase (Gururani et al., 2012), genes involved in the production and transformation of secondary metabolites (Erb and Kliebenstein, 2020), such as certain amine- and polyamine-oxidases (Cona et al., 2006), and components of the ubiquitination and proteasome complexes (Dielen et al., 2010). All these genes are present with the same copy number in both genomes. The only exception is one poly-amine oxidase gene, that is present only in PN40024 locus.

## 5 Conclusions

In this work, GWAS was used to identify loci associated with the resistance to grapevine DM and PM. The analysis allowed the discovery of two new loci associated with *P. viticola* resistance and two loci associated with *E. necator* resistance, in a population of *V. vinifera* varieties coming from one of grapevine domestication centers. The four loci were found to be located in chromosomal regions enriched with genes involved in different mechanisms of defense against biotic stresses, suggesting promising bases for their future exploitation in breeding programs of resistant varieties. To use them with the most efficient gain, further functional characterization studies could be performed, employing approaches such as Clustered Regularly Interspaced Short Palindromic Repeats (CRISPR) based systems or RNA interference technologies.

Employing resistance sources from *V. vinifera* germplasm could confer a great advantage to breeding programs, compared to using non-*vinifera* species. This is because they make it possible to obtain crosses with cultivated varieties in a faster way, while maintaining a good resistance level against specific pathogens. Moreover, they can provide a product free from the unpleasant characteristics typical of wild species crosses. The development of resistant grapevine varieties with high oenological potential not only leads to a reduced dependence on chemical interventions but also promote a transition towards environmentally friendly vineyard practices, resulting in decreased production costs and ultimately fostering greater sustainability within the wine industry.

## Data availability statement

The datasets presented in this study can be found in online repositories. The names of the repository/repositories and accession number(s) can be found in the article/**Supplementary Material**.

## Author contributions

VR: Data curation, Formal analysis, Methodology, Writing – original draft. MC: Data curation, Formal analysis, Methodology, Writing – original draft. GM: Data curation, Formal analysis, Methodology, Writing – review & editing. DMi: Data curation, Formal analysis, Methodology, Writing – review & editing. LB: Conceptualization, Funding acquisition, Writing – review & editing. DMa: Resources, Writing – review & editing. OF: Conceptualization, Funding acquisition, Resources, Writing – review & editing. ST: Data curation, Formal analysis, Methodology, Writing – original draft, Writing – review & editing. GD: Data curation, Formal analysis, Methodology, Writing – original draft, Writing – review & editing.
