# Supplementary material for: Novel loci associated with resistance to downy and powdery mildew in grapevine
Source: Front Plant Sci. 2024 Mar 22;15:1386225. doi: 10.3389/fpls.2024.1386225 (PMC10998452; doi:10.3389/fpls.2024.1386225)
Supplement: Supplementary file 1 [file DataSheet_1.docx]

Supplementary Material

# Supplementary Figures and Tables

## Supplementary Figures


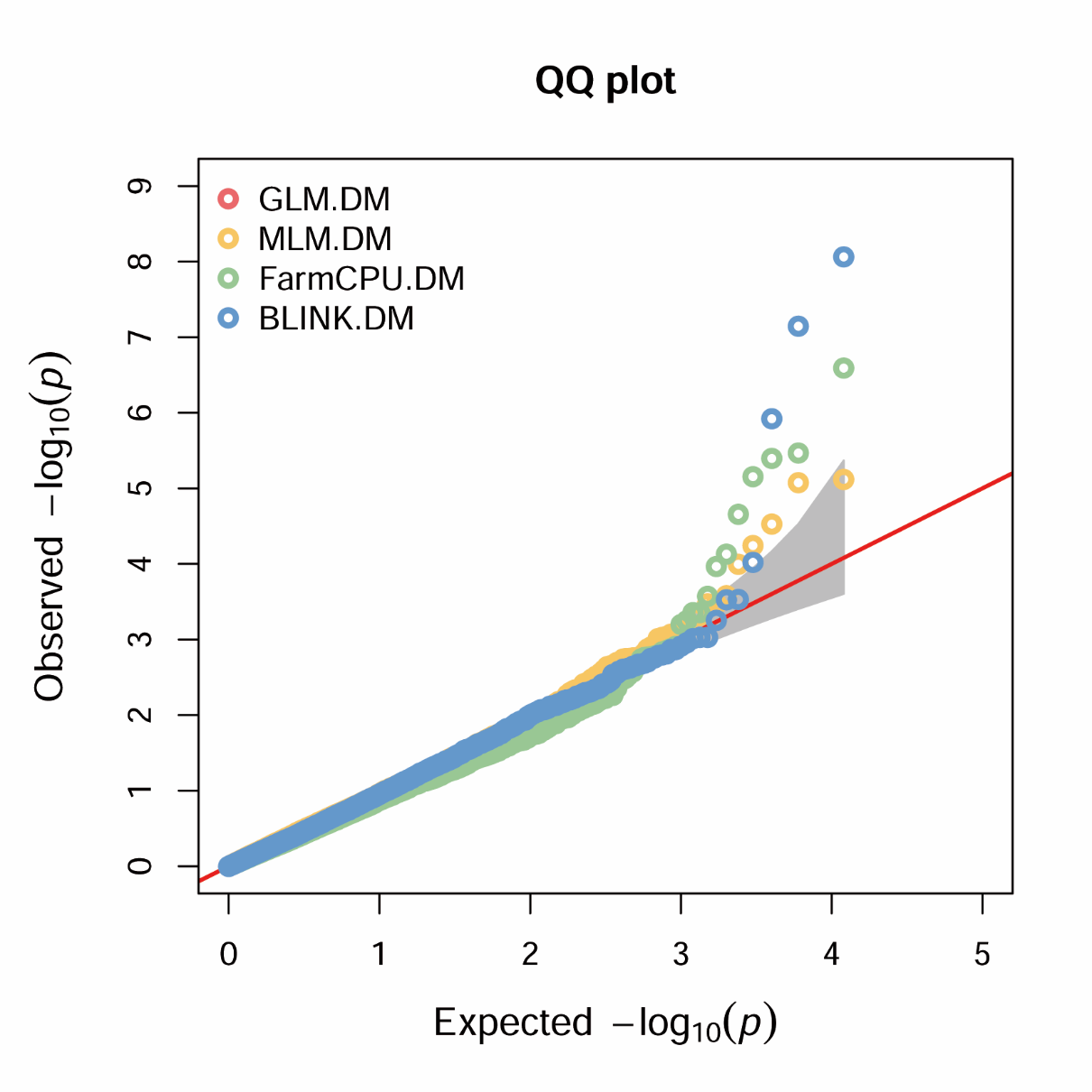


**Supplementary Figure 1.** Quantile-quantile (QQ) plot of expected vs. observed -log10 p-values estimated for binary (resistant vs. susceptible) coded phenotypic response to downy mildew (DM) in a panel of 88 cultivated and wild grapevine accessions genotyped by 18 k SNPs. Results of BLINK, FarmCPU, MLM and GLM association models.


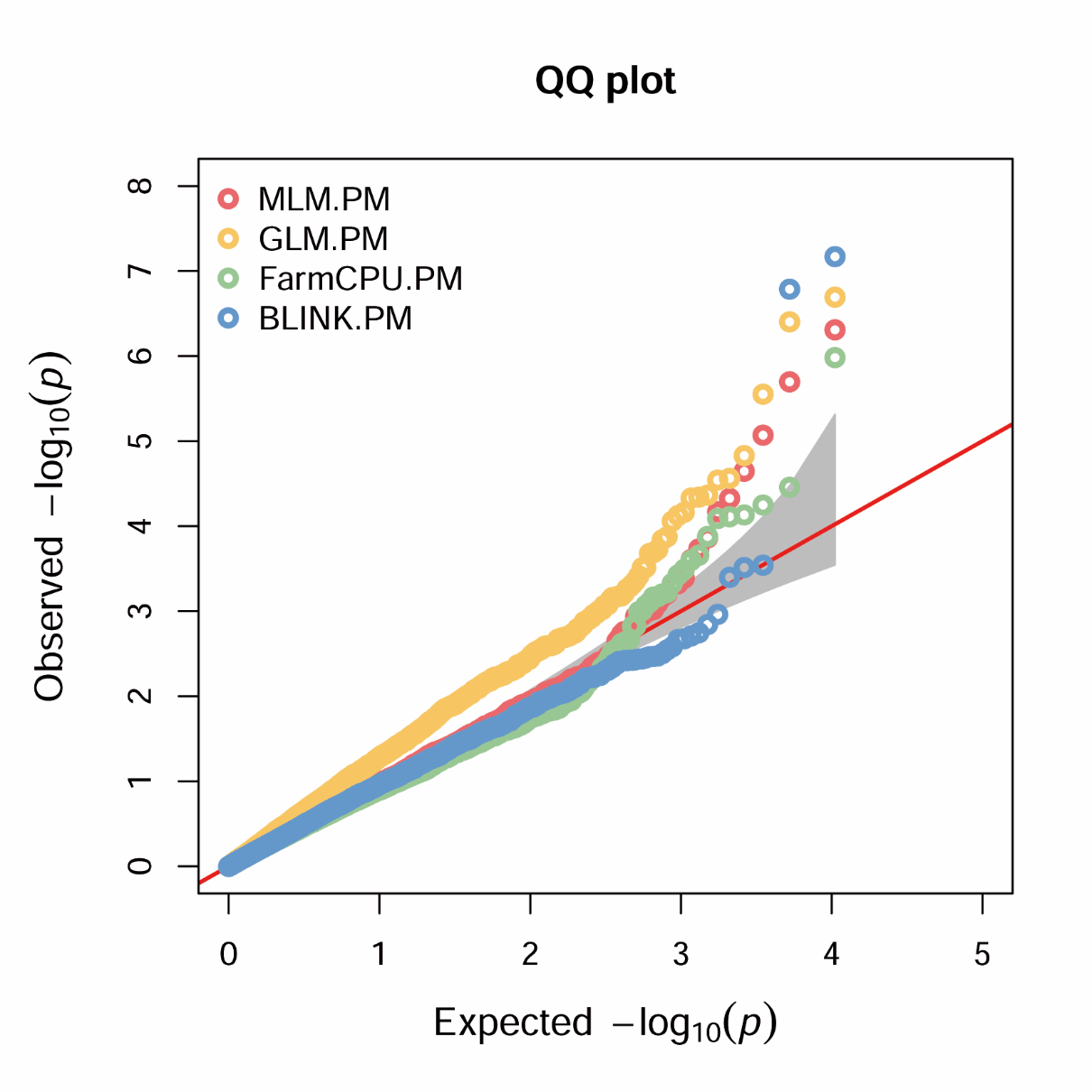


**Supplementary Figure 2.** Quantile-quantile (QQ) plot of expected vs. observed -log10 p-values estimated for binary (resistant vs. susceptible) coded phenotypic response to powdery mildew (PM) in a panel of 88 cultivated and wild grapevine accessions genotyped by 18 k SNPs. Results of BLINK, FarmCPU, MLM and GLM association models.

## Supplementary Tables

**Supplementary Table 1.** List of 88 grapevine genotypes, cultivated and wild, coming from Caucasus, Iran and Uzbekistan and SSR profiles. VVS2, VVMD5, VVMD7, VVMD25, VVMD27, VVMD28, VVDM32, VrZAG62, VrZAG79, ISV2, ISV3, ISV4 and VMCNG4b9 loci were used for varietal identification. Ren1, Rpv1-Run1 and Ren3-Ren9 loci were amplified for identification of alleles associated with resistance to downy and powdery mildew.

**Supplementary Table** **2.** SNP profiles of 88 grapevine genotypes, cultivated and wild, coming from Caucasus, Iran and Uzbekistan, genotyped at 18 k loci.

**Supplementary Table** **3.** List of grapevine accessions, cultivated and wild, coming from Caucasus, Iran and Uzbekistan, analyzed in this study. Information about subspecies, origin and resistance/susceptibility to *P. viticola* and *E. necator* are reported. 1: resistant; 0: susceptible.

**Supplementary Table 4.** Ancestry values at K = 3 detected for SNP profiles of 88 grapevine accessions (wild and cultivated from Caucasus, Iran and Uzbekistan), genotyped at 18 k loci.

**Supplementary Table 5.** List of candidate genes in a window of 0.5 Mb upstream and downstream the four SNPs associated to *P. viticola* and *E. necator* resistance trait. Genes have been identified on V. vinifera reference genome (PN40024v4) and Mgaloblishvili genome (haplotype 1 and haplotype 2).
